# Ethnomedicinal uses of *Hagenia abyssinica *(Bruce) J.F. Gmel. among rural communities of Ethiopia

**DOI:** 10.1186/1746-4269-6-20

**Published:** 2010-08-11

**Authors:** Biruktayet Assefa, Gerhard Glatzel, Christine Buchmann

**Affiliations:** 1 University of Natural Resources and Applied Life Sciences, Department of Forest and Soil Sciences, Institute of Forest Ecology, Peter Jordan-Strasse 82, 1190 Vienna, Austria; 2 University of Natural Resources and Applied Life Sciences, Department of Sustainable Agricultural Systems, Division of Organic Farming, Working Group: Knowledge Systems and Innovations, Gregor-Mendel-Strasse 33, 1180 Vienna, Austria

## Abstract

Ethiopian communities highly depend on local plant resources to secure their subsistence and health. Local tree resources are exploited and used intensively for medicinal purposes. This study provides insight into the medicinal importance of *Hagenia abyssinica *as well as the degree of threat on its population. An ethnobotanical study was carried out to document medicinal uses of *Hagenia abyssinica *by rural communities of North and Southeastern Ethiopia. The study was conducted using an integrated approach of group discussions, observation, a local market survey and interviews. A total of 90 people were interviewed among whom elderly and traditional healers were the key informants. Societies in the study sites still depend on *Hagenia abyssinica *for medicine. All plant parts are used to treat different aliments. Tree identification, collection and utilization were different among the studied communities. In spite of its significance, interest in utilizing flowers of *Hagenia abyssinica *as an anthelmintic seems to be diminishing, notably among young people. This is partly because the medicine can be harmful when it is taken in large quantities. Nowadays, the widely used *Hagenia abyssinica *is endangered primarily due to various anthropogenic impacts. This in turn may become a threat for the associated knowledge. It is recommended to assist communities in documenting their traditional knowledge. Measures for conserving species are urgently needed.

## Introduction

Plants have played a vital role in the prevention and treatment of disease since prehistoric times. People in different parts of the world depend on plant resources for their basic needs and are aware of many useful species occurring in their ecosystem. They have continuously developed their knowledge of traditional plant uses and plant resource management [1-4]. Traditional knowledge is described as 'a cumulative body of knowledge, practice and belief, evolving by adaptive processes and handed down through generations by cultural transmission, about the relationship of living beings (including humans) with one another and with their environment' [2]. In many countries of Africa, Asia and Latin America people depend on traditional knowledge and medicinal plants to meet some of their primary health care needs. For instance in Africa up to 80% of the population use traditional medicine for primary health care [5]. Likewise, many Ethiopian communities are dependent on local plant resources for medicine. Ethiopia is endowed with diverse biological resources due to significant geographical diversity, which favored the formation of different habitat and vegetation zones. Ethiopia is also home to a diverse mix of ethnic, cultural and linguistic groups. This diverse combination of social and cultural backgrounds contributed much to the existence of rich indigenous knowledge, including managing and using medicinal plants against human and livestock ailments. Plants have been used as a source of medicine in Ethiopia for a long time. More than 80% of the Ethiopian people are dependent on plants for their health service [6]. More than 95% of traditional medical preparations in the country are of plant origin [7]. Medicinal plants and knowledge of their uses provide a vital contribution to human and livestock health care needs. The importance of medicinal plants to treat human and livestock ailments in most parts of Ethiopia is stated by various authors [8-14].

**Figure 1:**
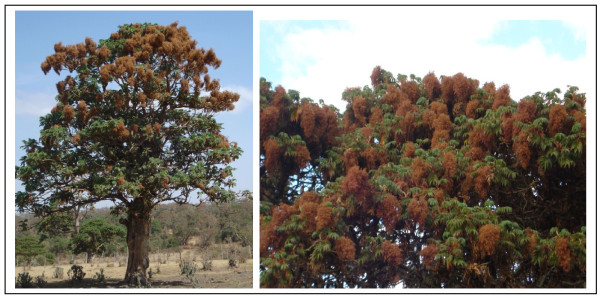
**Standing *Hagenia abyssinica *tree and its flowers**.

*Hagenia abyssinica *(Bruce) J.F. Gmel. is an important medicinal plant that societies relied on for generations for combating various ailments. *H. abyssinica *is a multipurpose dioecious tree in the plant family of Rosaceae. It is a tree growing up to 20 m (Figure 1). The species also occurs in Kenya, Tanzania, Uganda, Sudan, Congo, Malawi, Burundi and Rwanda. *Hagenia *has been used as a remedy for intestinal parasites, especially against cestodes [15]. It has served as an anthelmintic in ruminants [16] and also against tapeworms in humans [17,18]. Besides being a source of medicine, *Hagenia *has been utilized for various other purposes such as construction, furniture, fuelwood, and soil fertility management. As a result of its enormous significance, *H. abyssinica *is one of the endangered tree species in the country due to overexploitation [19]. Accordingly, the Forestry Law [20] prohibits the utilization/harvesting of *Hagenia abyssinica *. The proclamation was enacted with a view of providing and enhancing better conservation, development and utilization of forests. However, in practice, there is a lack of law enforcement. Consequently *H. abyssinica *population is increasingly endangered.

Habitat destruction reduces the existence of such important species, and thus negatively affects several aspects of human well-being, such as food security, medicine as well as the associated traditional knowledge. In spite of its significance, information on the traditional use of *H. abyssinica *has experienced little consideration. Studies have been conducted on medicinally important Ethiopian plant species among which *H. abyssinica *is also listed [14,18,19,21]. However, former studies do not provide sufficiently detailed information on the utilization of *H. abyssinica *; hence further research is necessary. This study aims to examine in detail the traditional knowledge on the identification, harvesting, preparation and utilization of *H. abyssinica *by Ethiopian rural communities. The medicinal value of *H. abyssinica *to the rural communities is highlighted. Factors influencing current utilization rates are identified and current management and conservation strategies examined. The first section provides an overview on description of the study sites and the methods used to document traditional knowledge. Traditional *H. abyssinica *processing and uses of various plant parts for medicinal use are described in the second part. In the concluding remarks the importance of *H. abyssinica *, as well as the degree of threat on its population and local conservation efforts are highlighted. Suggestions to promote sustainable utilization of *Hagenia abyssinica *are presented.

## Methodology

### Study areas

**Figure 2:**
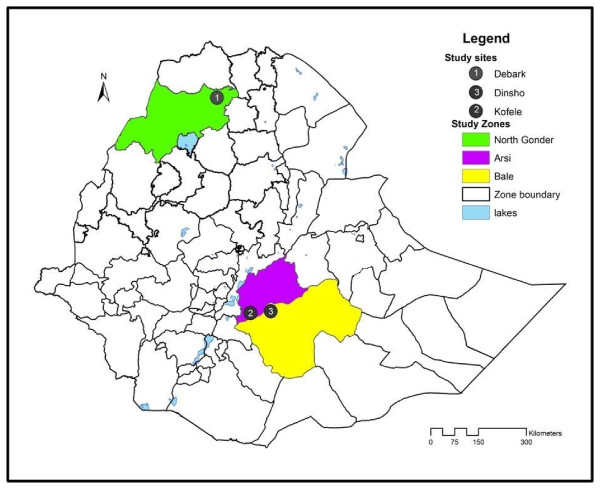
**Location of the study sites**.

The study was conducted in three different parts of the country (Figure 2). The study sites were chosen systematically so as to conduct both ecological and social studies on *Hagenia abyssinica *. The social part is presented in this paper while the ecological field data will be published in a separate paper. The study site 'Milligebsa' (hereafter described as Debark) is located in Amhara Regional State, Northern Ethiopia which is in 18 km distance from Debark town. The geographic location is 13°11' N and 37°58' E. The altitude ranges between 2800 m-3150 m a.s.l. The mean annual rainfall ranges between 900-1400 mm. The minimum temperature usually drops to -3°C and -5°C at night. Soil type is characterized as Haplic Cambisols of silty clay texture. The people in the study site are *Amharas *who are engaged with subsistence farming and livestock rearing. *Amharic *is the language spoken. The second study site is a small village called 'Deyu' (hereafter described as Kofele) which is located in Oromiya National Regional State, Southeastern Ethiopia. It is in 5 km distance from Kofele town. It is located between 7°11' N and 38°52' E. The area lies between 2600 m and 2750 m a.s.l. Annual average rainfall is about 1232 mm with a mean monthly rainfall of 102.6 mm. The mean monthly minimum and maximum temperature is about 5.4°C and 19.8°C respectively [22]. The soil is characterized as Haplic Luvisols of clay texture. Vegetation is mostly composed of perennial grasses and tree species. The *Oromos *are the dominant people residing in the area, among other ethnic groups. *Oromiffa *is the local language spoken. Most are small holder subsistence farmers engaged with agriculture and animal rearing.

The third study site 'Dinsho' (hereafter described as Bale) is located in the Bale Mountains National Park (BMNP). It is also set in Oromiya National Regional State, in Southeastern Ethiopia, and lies within the geographical coordinates of 7°06'N and 39°47'E. The altitude lies about 3200 m a.s.l. The area has a bimodal rainfall characterized by a rainy season lasting from March to October and a dry season that extends from November to February [23]. Mean annual rainfall is about 1218.6 mm. The mean annual minimum, and maximum temperature is 2.4°C and 15.5°C, respectively. The present topography is a reflection of long term weathering processes originating from Oligocene lava outflows [24]. Soil in the study site is characterized as Mollic Andosols with silty clay texture. The *Oromos *are the dominant ethnic group living in the area, among other ethnic groups. *Oromiffa *is the local language spoken. Main economic activity is based on mixed farming which involves pastoralism and cultivation of crops.

### Data collection and analysis

Prior to data collection, a preliminary field survey was carried out in March 2008. The intensive data collection was subsequently conducted in three phases. Communities in the Kofele area were interviewed in the1^st ^phase, from April to May 2008. The 2^nd ^phase (June - July 2008) was allocated to communities living in the Bale Mountains National Park. Communities in Debark area were interviewed during the 3^rd ^phase (August-September 2008). Methods used to document the traditional knowledge included interviews, observation, and open group discussions with local communities. In addition, a local market survey was conducted. A total of 90 people (30 from each site) were chosen systematically following [25]. Informants were chosen with the help of elderly people and local administrators in the study sites. Semi-structured interviews were conducted following [26]. At Bale and Kofele sites the interviews were conducted in the *Afaan Oromo *language. Though the corresponding author understood the language at times a local translator assisted in the interview process. The interviews in the Debark site were conducted in *Amharic *language. Each informant was interviewed separately and advised not to discuss with each other so that they could provide independent information. Interviews were conducted in places such as school compounds, providing a comfortable space to all. Before conducting the interviews, informants were briefed about the aims of the study and gave prior informed consent. Where applicable the International Society of Ethnobiology (ISE) code of ethics [27] were respected. Questions were asked in a stepwise manner by first asking relevant data on their age, sex, address, level of education and occupation. Following to that, informants were asked to share their knowledge on the utilization of *Hagenia abyssinica *. This included: how long and for what purpose they have been using *H. abyssinica *, plant parts used, preparation methods, form used (fresh/dried), mode of application, as well as identification, collection and utilization. Respondents were asked to state the status/degree of scarcity of the species, factors affecting the current utilization rate and if there were any management and conservation activities taking place in the area. In addition, observation and in-depth interviews with key informants, such as elderly and traditional healers, formed part of the field research. As this study is conducted along with an ecological study of *H. abyssinica *a number of plant species were encountered in the study areas. All plant specimens including those mentioned in the present study were identified at the National Herbarium, Addis Ababa University, where voucher specimens were deposited. Data were organized in Excel (Microsoft 2003) datasheets. Responses given by respondents were coded into numerical form for the analysis (e.g. 1 = Yes, 2 = No) and simply presented as percentages.

## Results and discussion

### Tree identification

Informants in all sites knew *Hagenia abyssinica *, many of them since their childhood. Hagenia is a dioecious species with separate male and female trees that are identified under different local names (Table 1). Trees with bright pinkish-red inflorescence and bulkier flower heads are considered to be female and the ones with yellowish color and feathery flower heads are regarded as male trees [28,29]. About 13 respondents in Debark (7 male respondents), 20 in Bale (17 male), and 24 in Kofele (13 male) site stated that they are able to differentiate between male and female trees.

**Table 1:** Local name of male and female *Hagenia abyssinica *trees

Study site	Local language	Local name of *Hagenia abyssinica*	Local name for male tree	Local name for female tree
Kofele	Oromiffa	Heto	Balfe	Hatiya
				Artu

Bale	Oromiffa	Heto	Gurumbo	Degemele
			Korma	Hache
				Artu

Debark	Amharic	Kosso	Wende	Sete

### Plant part collection

Informants stated different times as the 'best time' for plant collection. In Kofele for example, the best time to collect the inflorescence is from October to February; while in Bale it is from October to end of January. People in Debark site collect in November and December. Collection during these months is interrelated with the fruiting and flowering phenology of *H. abyssinica *. Apart from the inflorescences, collection of other plant parts (e.g. root, bark) can be carried out any time. Respondents further stated that though it is possible to collect plant part at any time of a day, but it is more preferable to do it in the morning. This is associated with the effectiveness of the medicine. Sixty percent of respondents in Kofele, 42% in Bale and 22% percent in Debark site explained that parts of *H. abyssinica *collected in the morning time could have strong and effective medicinal properties to treat any ailment. Common technique to collect plant parts includes climbing the tree, which is usually done by children. Leaves on lower branches, as well as pieces of bark, are gathered by hand while roots are collected by digging. People in Bale and Kofele areas used forked branches of trees and bamboo sticks to collect the inflorescence part. In the same study site people carefully cut the tree trunk to obtain the sap. In Debark people use *Kezera *(a walking stick) for collecting the inflorescences. The amount of plant parts collected by the people depends on the uses. A family collects a small quantity if the use is aimed for domestic purposes. For example, to prepare a self-made remedy against intestinal ailments, a small amount of plant parts (mostly the inflorescence) is collected. Alternatively, several sacks could be collected if intended to be sold on the local market.

### Plant utilization

Utilization of *H. abyssinica *was stated as significant to the communities of all study sites. This confirms its considerable value to different societies of the country which is also stated in the literature [9,13,29,35]. This particular study confirms that parts of *H. abyssinica *are used against several human and livestock ailments. Medicinal uses were categorized as intestinal, digestive, circulatory, respiratory and nervous system, among others, disorders (Table 2). The anthelmintic action of *H. abyssinica *against tapeworm (*Taenia saginata *Goeze), whose widespread occurrence grounds in the consumption of dishes containing raw beef, has been mentioned by all informants in all study sites. Hagenia has been also described as a powerful remedy for intestinal parasites, especially against cestodes [10,11,14,15,17,30-32]. In the 19^th ^century, the species was included in most European pharmacopoeias as an effective drug against intestinal worms, which made it one of the most famous African plants at that time [33,34]. In addition to its importance against human ailments *H. abyssinica *has anthelmintic property to treat ruminants such as cattle, goats, and sheep. Its bark and leaves are used to treat livestock diseases. Informants in Bale and Kofele explained that a decoction of bark is given to cattle and equines to treat a disease that turns livestock thin and skinny. In Debark, fresh leaves of *H. abyssinica *are wrapped on fractures of equines. Medicinal uses of *Hagenia *against livestock ailments have been also documented in literature [14,16,30,35].

**Table 2:** Medicinal value of parts of *H. abyssinica *in the study sites

BALE				
**Bark**	**Flower**	**Root**	**Leaf**	**Wood**

Fever/cough	Intestinal worms (tape worm)	Stomachache	Diarrhea	Stomachache (reddish color liquid from the sapwood)

Stomachache	For healing wound		Typhoid	

Cold (bronchitis)	Epilepsy*		Cough	

Livestock disease (thin/skinny body)	Evil eye		Livestock disease (mixed with *Juniperus procera*)	

**KOFELE**				

**Bark**	**Flower**	**Root**	**Leaf**	**Wood**

Dermatology	Intestinal worms (tape worm)	Stomachache	Diarrhea	Stomachache (reddish color liquid from the sapwood)

Malaria	Hepatitis*	Severe abdominal pain	Livestock disease	

Stomachache	Sexually Transmitted Diseases (STDs)	Throat disease		

Livestock disease	Problems related to Bile	Cancer (mixed with other plants)*		

**DEBARK**				

**Bark**	**Flower**	**Root**	**Leaf/seeds**	**Wood**

Livestock disease	Intestinal worms (tape worm)	Severe stomach pain	For healing injured part (human/livestock)	

* Information obtained from traditional healers

### Mode of preparation

In general, plant parts of Hagenia are processed either in fresh or dried forms. Children or elder people collect the plant part which is intended for remedy preparation. Usually elderly men are responsible for harvesting the bark and root part. The process of preparing medicine from female flowers (here after called as *kosso *) is simple yet requires care. Normally the name *kosso *refers to the tree itself (in Amharic), the human tapeworm (Taenia saginata Goeze) or the medicine. *Kosso *preparation comprises different steps, and considers details like which part needs to be used, amount and substances to be mixed (if any), and time to prepare the solution. For the preparation the flower is sun-dried so that it can easily be separated from the whole inflorescence. In Bale, the whole inflorescence is covered for 1 or 2 days with animal skin (pelt usually from a cow or an ox) or with leaves of *Discopodium penninervium *Hochst, and later kept in the sun for further drying. After drying the flower is roasted on an iron plate and pounded using a pestle and mortar. Traditional stone grinders are also used in all of the study sites. Usually women are responsible for this job. A small amount of dried flowers is ground into a powder and then sieved. Afterwards the fine powder is kept in a bowl. In Bale, women keep the powder in traditionally made materials such as *Chocho *(milking pail), *Tunto *or *Kila *(type of bowl usually made to keep butter). Eventually, the fine powder is mixed with different substances (Table 3) and then consumed. In Kofele and Bale areas the preparation of *kosso *is usually carried out during the night. In these sites the remedy has to ferment for some time. The shortest possible fermentation time usually takes 1-2 nights. However, in places like Bale it can be kept for about 10 days due to the cooler climate. Informants noted that keeping the remedy for a longer time helps to reduce bitterness and increase the effectiveness of the medicine. On the contrary, the medicine was prepared early in the morning and instantly consumed in Debark. On average, the preparation takes 30-60 minutes.

**Table 3:** Substances mixed with powdered flowers of *H. abyssinica *(*Kosso *medicine)

Substance	Study site
Cold water	A, B, C
Warm water	A, B, C
Milk	A, B
*Arera *(sour defatted milk)	A, B
*Aguwat *(whey)	A, B
Honey	A, B, C
Banana	A, B, C
Kebericho (*Echinops kebericho *Mesfin)	A
Dobi (*Girardinia bullosa *(Steudel) Wedd.)	A, B
Hinkoko (*Embelia schimperi *Vatke)	A, B, C
Cabbage (*Brassica oleracea *L.)	A
Pumpkin (*Cucurbita pepo *L.)	A

**Key**: A = Bale; B = Kofele; C = Debark

### Means of application

In all of the study sites, *kosso *is usually taken orally in the form of a decoction. However, it can also be consumed in the form of paste (e.g. by mixing the powdered flower with banana or honey). Usually children and very weak patients prefer to swallow the sweet paste as the medicine is bitter. Respondents in Bale mentioned that *kosso *can be consumed with Dobi warabechaa (*Girardinia bullosa *(Steudel) Wedd), or pumpkin (*Cucurbita pepo *L *.) *seed, or cabbage (*Brassica oleracea *L *.) *or 'Hinkoko' (*Embelia schimperi *Vatke). Fruits of *Embelia schimperi *Vatke are usually grinded, macerated in water and mixed with the already prepared *kosso *solution. Another ethnobotanical study conducted in the region [29] reported that people in 'Dheeraa' town, Ethiopia, mix a pounded flower of *Hagenia abyssinica *with the root of *Croton macrostachyus *Hochst. ex Del. or leaf of *Grewia ferruginea *Juss. for worm expulsion. Respondents in the present study sites believe that mixing the two medicines could result in great medicinal effect against taeniasis, help reduce bitterness of the medicine as well as nausea. Informants in Debark also added that *kosso *(in paste form) can be taken with *Kita *, a kind of bread which is made from unleavened dough of *Teff *(*Eragrostis teff *(Zucc.) Trotter).

All respondents stated that *kosso *needs to be consumed in the morning on an empty stomach. The patient is not allowed to eat after taking the medicine until the proglottids (segments) are expelled from the intestine. They further noted that *kosso *medicine would be more effective when the proglottids are made to starve. Preventing a patient from eating for sometime is also mentioned [36]. Informants in the study sites stated that after having consumed *kosso *, people usually experience nausea, stomach/abdominal pain and continuous diarrhea which may last for about 6 hours, and these side effects are considered to be normal in the community. Usually after 4 to 5 hrs segments of the worm are expelled. The patient is then provided with a warm meal, such as porridge, meat soup or *Shiro *(sauce made of powdered peas (*Pisum sativum *L.) with 'Injera' (thin flat bread made of *Teff *cereal). All respondents in Debark stated that by no means is the patient allowed to drink *tella *(locally made beer), because the interaction reduces the effectiveness of the medicine. Societies in Debark believe that *kosso *medicine would be effective if it is prepared by a virgin girl, a sterile woman or menopausal woman in her menopause. Apart from oral intake, dermal application is also practiced in Debark site.

Currently, *kosso *medicine is being utilized by the communities of all study sites. Forty three percent of respondents in Bale, 40% in Kofele and 30% in Debark explained that this self-made remedy is widely accepted within the society as its hygienic effect gives great psychological satisfaction. Nevertheless, the prevalence of gastrointestinal upset after consumption is forcing people to reduce the intake or to change the means of preparation. Women actively take part in updating the traditional knowledge and try out different methods to reduce the side effects associated with drinking *kosso *. Female respondents in Bale explained that they consume *kosso *in the form of a decoction. A small amount of powdered flower is boiled and served in the form of tea. Another means of application included the utilization of bark in the form of smoke. Respondents in Kofele and Bale areas explained that during post partum period, mothers smoke the bark by heating it over the fire. Female respondents further stated that the practice eases muscle aches and stiff joints, stimulates blood circulation and boosts the immune system. In addition, people applied the powdered seeds on wounds and cuts of both humans and livestock.

### Prescription dosage, side effects and remedies against side effects

The majority of informants explained that both self-made, and medicines prescribed by healers need to be taken in specific doses, but they stated different amounts. Common measurement units mentioned in all communities include tea cups, water glasses and cans. Respondents explained that the dosage depends on age, sex, physical appearance of a person, health condition and severity of pain. Similar studies have also mentioned such measurements [8,36-39]. Eighty five percent of respondents in Bale, and all informants in Kofele and Debark believe that the dosage usually depends on age and sex. For instance, the smallest portion of *kosso *solution (< 250 ml) was usually given to very old people as well as to young boys and girls (~250 ml). In general, men consume the largest portion (500 ml to 750 ml) followed by women (300-500 ml). Even today, pregnant women in Bale and Kofele areas continue to drink *kosso *. All female respondents in these two sites explained that there was a tradition to take very small amounts (~200 ml). Usually when a woman starts her 3^rd ^or 4^th ^month of pregnancy she drinks *kosso *. Some of them continue till the 8^th ^month. There are also cases when a woman drinks shortly before delivery time. The women believe that it helps in reducing pain during delivery, and improves the health of both baby and mother. A health condition of a patient is another aspect considered before taking the medicine. They further stated that comparatively an extra amount is provided for a very ill person. In former days, it was compulsory for children to drink *kosso *once a month when they reached at the age of 7 or more (in Bale), 12 or more (in Kofele), and 10 or more (in Debark). The reason was by this time the children have already started eating raw meat. Overall, no age guideline is used as standard, but it is merely a decision made by the family.

Usually, medicines have some side effects, and self-made ones are no exceptions. Informants in all study sites stated that severe stomach pain accompanied by diarrhea and occasional nausea were the usual side effects of *kosso *medicine. The frequency and seriousness varied greatly from patient to patient. A person may also suffer from muscular tiredness, fatigue and could even faint, if taking an overdose. The administration of an overdose may be linked to a belief that extra consumption could result in an enhanced medicinal effect; however, it has been reported that blindness and changes in the central nervous system function have been found in people who took an overdose of *Hagenia abyssinica *[40]. It is also reputed to cause abortion in women [41,42]. Excess dosage of *kosso *medicine can even cause death [41]. Despite widespread use as an anthelmintic, few reports noted that *kosso *medicine was found to be toxic [43]. In the present study, informants also mentioned the negative effect of *kosso *such as gastrointestinal upset, however, if serious problems occur due to an overdose, the person is usually provided with a remedy to counteract the side effects. This could include boiled milk, yogurt, coffee or water. In Bale and Kofele a patient was given a porridge made out of barley (*Hordeum vulgare *L.) flour, and a soup made of oat (*Avena sativa *L) or barley. Elders in Bale explained that if the pain was severe the patient was supplied with fried mutton or sheep liver. In Debark, a soup prepared from flax seeds (*Linum usitatissimum *L.) was usually given to the patient. The seeds were first roasted, pounded, and then boiled.

Regarding the current utilization of *kosso *the majority of respondents (57% in Bale, 60% in Kofele and 70% in Debark) stated that they are currently not utilizing *kosso *medicine. Of this percentage, 94% in Bale, 78% in Kofele, and 81% in Debark were male respondents. This is primarily due to an increase in awareness of side effects from unknown dosage. Introduction of modern medicines and the declining of *H *. *abyssinica *population in the forest also play a great role. For instance, all informants in the Debark site stated that the tradition of drinking *kosso *still exists, but it becomes more and more difficult to collect the inflorescence as today few scattered trees exist in the area. In addition, people have realized the advantage of taking modern medicines that don't have many unwanted effects that also help them continue with their daily task without serious side effects. Respondents reported that the introduction of modern medicine has affected the value of traditional medicines in general. Low prices persuaded people to buy broad-spectrum drugs from local drug shops; however, the short supply of such drugs is a problem. Niclosamide, Mebendazole and Albendazole were among the common medicines with anthelmintic properties. These drugs have different dosages and are given to a patient based on age and/or weight. The price was about 2 to 4 ETB (equivalent to 0.18 to 0.36 USD) at the time of the field study. While conducting this study, people in Kofele area were seen buying these medicines from local drug shops. Most of them were youngsters, and they explained that modern medicines don't have many side effects and are simple and ready to be used. Moreover, those medicines don't disturb their daily work. They further highlighted that traditional medicines have not been scientifically studied or verified. They believe that traditional healers give medicines by trial and error. Traditional medicines sold on local markets were not effective due to inappropriate handling, and were mainly based on false information. Due to these reasons they prefer modern medicines over traditional ones.

### Traditional knowledge and its transfer

In most developing countries, including Ethiopia, traditional knowledge on the medicinal uses of plants has been passed down from generation to generation as part of an oral tradition. Informants in all study sites stated that elder men usually share their knowledge with one of their sons, most often to the first-born. This particular son may be chosen because the father loves him very much or the son is especially keen and interested in traditional medicine [44]. This was mentioned by all male informants. Female respondents however explained that they prefer sharing their knowledge with their daughters. Traditional healers play a key role in transferring traditional knowledge in the society. Respondents explained that in need of help, it is customary to visit a traditional healer. People visit healers because they believe that healers have good knowledge of traditional medicines. In the study sites almost all of the healers were elder men. Informants explained that there are very few female healers in the area. The stated reason was that women have less interest in the practice. Men are reported to take it seriously, and for some it is even used as a base for supporting their family. Moreover, the studied communities believe that male healers have better knowledge than women, and hence the medicine prescribed by them is perceived to be more powerful. Such believes have resulted in the transfer of traditional knowledge mostly between the healer (men) and his children (mostly sons). In the present study sites even though female informants were few in number they were observed to have a higher level of knowledge than men regarding the detailed processing of parts of *H. abyssinica *for different medicinal purposes, especially against intestinal diseases.

By and large, in all of the study sites interest towards utilizing traditional medicine is diminishing among the younger generation. Some of the reasons mentioned include: the tendency to modern education, the migration to cities for profitable jobs, the decline of the medicinal plant population due to deforestation, and the introduction of modern medicines. Similarly, some elder people were becoming reluctant to take traditional medicines when they have already experienced severe side effects. Due to these factors, the practice is now becoming more and more outdated. This is also mentioned in another study [29]. Thus, many individuals are not willing to share their information with their children, except the knowledge related to livestock medicines. In the present study more information was obtained from elderly informants than the young ones. This could indicate a lack of interest which ultimately results in loss of knowledge. Similar studies conducted in the country also support such findings [9].

### Market condition

Even though the population of *Hagenia abyssinica *is no longer as abundant as before, its medicine is still available in local markets for a low price. Dried flowers were commonly found in all markets of the study sites. In Bale a quarter of a kilo of dried flowers was sold for about 1 ETB (equivalent to 0.1 USD). Usually small plastic cups or cans are used to measure the quantity. For one quintal the maximum price could reach up to 60-70 ETB (5-6 USD). Sometimes it may even reach up to 100ETB (9 USD). Income derived from this sale is of particular importance to the poor households, especially for women, in meeting their basic needs such as food. In Kofele market, women in their 50's were observed selling the dried flowers of *Hagenia abyssinica *. They explained that in the former days it was common to barter for two cups of dried flower with one cup of cereal crops like barley or wheat. In general, women, especially those who are married, or elders, were major vendors in the market. Unlike elder men young children help their mothers in selling the medicine. In line with this, healers also explained that they prepare medicine using different parts of *H. abyssinica *and its contribution to their income is considerable. Marketability of *H. abyssinica *on local markets was also mentioned in [30].

### Current population of Hagenia abyssinica and Conservation activities

In Ethiopia the *H. abyssinica *population has drastically decreased due to the growing pressures from various anthropogenic factors. This study revealed that all informants in all of the study sites were aware of the scarcity of the species in their locality. Recalling their childhood times, they explained that there were plenty of *H. abyssinica *trees in the surrounding forests, but now the population has reduced significantly. Respondents in Debark site stated that the species is very scarce and only few scattered trees were left around their homes and nearby churches. Informants stated that an increase in human population, leading to settlement changes and land clearing for agriculture, was the main driving factor for the decline in the *H. abyssinica *population. Residential area expansion led to a significant loss of forest land as more people needed more lumber to build their houses. This ultimately created a big pressure on the surrounding forest. In addition, the extent of communities' involvement in agriculture seems to be increasing in all of the study sites. Informants categorized possible causes with the greatest impact on the depletion of *H. abyssinica *population as due to: heavy utilization i.e. selective cutting for (1) timber, (2) furniture, (3) house construction, (4) firewood use (5) medicine and (6) all other uses combined.

Heavy grazing and browsing impact from both livestock and wild animals was also mentioned as a reason that hampers the growth of young seedlings. Uncontrolled fire setting was another cause mentioned by informants. In many instances communities set fire to stimulate a new herbaceous growth to be used as livestock fodder but sometimes the fire burns trees and destroys large areas. In addition, trees may be killed by debarking, as this has been observed in the Kofele area. There, some *H. abyssinica *trees were found debarked. Reasons could be the extraction of bark for medicine or a systematic way of killing the whole tree. The effort to conserve the species in the study areas seems minimal. Although all informants have reported their interest, only few 30% in Kofele, 26% in Bale, and 7% in Debark sites have actually planted *H. abyssinica *trees in their home garden. They explained that they usually transplanted seedlings from nearby forests to their backyards. Management practices included watering, fencing and adding organic fertilizer to the seedlings. Conversely, the majority of informants (74% in Bale, 70% in Kofele and 93% in Debark) explained that even if they have the interest they were 'not able' to plant *H. abyssinica *trees. Lack of seedlings is one of the reasons for not planting. Although they were not capable enough to find as many seedlings as they would like to have some informants were able to collect and transplant wild seedlings from the forest but the seedlings did not survive due to frost and livestock pressure. In addition, the community usually prefer to plant fast growing exotic species such as Eucalyptus spp.

By and large, respondents in all study sites highlighted the importance of *H. abyssinica *in their community. Because of its role in the society, people in Bale and Kofele areas described *H. abyssinica *as 'Hangefa Muka' meaning 'one of the oldest and most respected trees'. However, its population is drastically declining, and therefore, they would like to plant seedlings and carry out appropriate management activities. Consequently, they call for support from agricultural bureau or any other development organizations to get seedlings. Moreover; they suggested that carrying out an extensive awareness creation effort in their localities is timely. Plantation activities carried out around Debark and Kofele sites indicate some awareness, but should be further intensified. Providing continuous care for seedlings and setting up of protective boundaries against livestock browsing should be encouraged.

## Conclusion

Communities in the study areas highly value *Hagenia abyssinica *for its medicinal properties. Though all parts of this medicinal plant are important to local communities, the most frequently used and mentioned part are the flowers, which carry anthelmintic properties, and are used against intestinal parasites (e.g. tapeworms). *Hagenia *is also used as veterinary medicine against many livestock ailments. Harvesting time, amount, purpose and prescriptions are found to be different among the study sites. Current utilization of *H. abyssinica *could be the result of a continued dependence of local communities on the species for their medicinal need. *H. abyssinica *is marketable thus provides the opportunity to raise household income. This study has shown that returns from selling mainly its dried flowers are important, particularly to the poor households. Even though *H. abyssinica *offers diverse products, the tree population is in decline due to anthropogenic factors. This study provides insight into the local importance of *H. abyssinica *as well as the degree of threat on its population. The scarcity of the species in the locality was mentioned by all respondents. People recall their past time experiences and compare those to the present day situation of local resource abundance. Recalling the vegetation cover of their sites informants in the studied areas (especially in Debark) strongly suggests that deforestation of *H. abyssinica *has been significant in the area. In all study sites, the extent of communities' involvement in agriculture was found to be high and seems to be increasing. This could result in more and substantial losses of *H. abyssinica *population which could ultimately lead to the fading away of the indigenous knowledge associated with the species.

Knowledge about identification, harvesting, preparation and utilization methods is still maintained within the community but in general, interest towards utilizing the traditional medicine is diminishing among many especially with younger people. Despite its widespread use *kosso *medicine is found to be harmful to health particularly when it is taken in large quantities. The prevalence of gastrointestinal upset following consumption is leading the majority of people in the study sites to reduce, modify way of intake or stop drinking completely. *Kosso *medicine is note taken by the majority of informants because of its side effects; some however (especially women and elderly people) still utilizing it. This may perhaps help to ensure the maintenance of knowledge on the species. In conclusion, it is useful to assist communities to document their knowledge. Moreover, averting illegal cutting and allowing natural regeneration of the population by protecting young seedlings from human and livestock destruction can help conserve this species. Creating public awareness and community-based management is timely and the current plantation activities that are carried out in the study sites should be further encouraged.

## Competing interests

The authors declare that they have no competing interests.

## Authors' contributions

BA and GG conceptualized and designed the study. The corresponding author collected field data and drafted the manuscript. CB participated in the enrichment of the manuscript. All authors read and approved the final manuscript.
